# Inequalities changes in health services utilization among middle-aged and older adult disabled people in China: based on CHARLS 2011–2018

**DOI:** 10.3389/fpubh.2024.1434106

**Published:** 2024-10-01

**Authors:** Shengxuan Jin, Ruobing Fa, Jiaqi Wu, Jiawei Lin, Shuyuan Zhang, Majid Ali, Shaofan Chen, Dongfu Qian

**Affiliations:** ^1^Jiangsu Provincial Institute of Health, Nanjing Medical University, Nanjing, Jiangsu, China; ^2^School of Public Health, Southeast University, Nanjing, Jiangsu, China; ^3^Center for Global Health, Nanjing Medical University, Nanjing, Jiangsu, China

**Keywords:** persons with disabilities, health service utilization, inequality, concentration index, longitudinal comparative study

## Abstract

**Background:**

Multiple intersections, including socioeconomic inequalities, influence health equity for disabled people and sub-populations. However, this association has not been sufficiently analyzed among Chinese-impaired persons. This study aimed to investigate the health services utilization and inequalities in middle and older adult persons with disabilities and subgroups.

**Methods:**

The China Health and Retirement Longitudinal Study (CHARLS) database in 2011, 2013, 2015, and 2018 were used. Health services utilization was measured by outpatient, inpatient, and self-treatment service utilization. Types of disabilities were classified into six categories. The pooled cross-section regression, concentration index, horizontal inequity index, and concentration index decomposition were used to evaluate inequalities and explore their main contributing factor.

**Results:**

The utilization and non-utilization of healthcare services showed variations across years (*p* < 0.05). The CIs and HIs for inpatient health service utilization were positive for all years and disability types. The total CIs of inpatient utilization were the highest (0.248). The highest disparities in utilization of inpatient services were for physical disabilities (0.4515 for CI in 2011), and the highest in self-treatment services were for intellectual disability (0.1538 for CI in 2011). The expenditure factor was the main contributor to inequalities. Chronic disease, educational level, and health insurance factors also contribute to the utilization inequalities.

**Conclusion:**

Policies should promote medical insurance and assistance for disabled people with serious impairment and poor. It is crucial to improve the provision of basic medical services, including meeting the demand for varied disabilities and the accessibility of facilities and equipment to enhance the access and well-being of people with disabilities.

## Introduction

People with disabilities are among the most disadvantaged and marginalized populations, frequently facing prejudice and challenges in terms of social, economic, and health disparities (1). Around 1.3 billion people, or 16% of the world’s population, were estimated to be disabled (2). China currently has the largest number of persons with disabilities in the world, with over 85 million individuals affected (6% of the country’s total population) (3). The general trend toward an increase in human life expectancy is leading to an aging population, which will inevitably lead to a significant increase in the number of older people with disabilities and semi-disabilities. Healthcare is crucial for people with disabilities as it provides equal opportunities by sustaining fundamental function and health promotion (4). A person with disabilities is more likely to report being in poor health and to have a higher incidence of chronic diseases such as diabetes, cardiac disease, overweight, and asthma (5–7). Compared to younger adults with disabilities, older adults with disabilities are more likely to have functional limitations and co-morbid conditions (8). Thus the demands for the use of healthcare services among those with disabilities are rising. However, the increased expenditures on health care, personal care, equipment, or other modifications connected to disabilities may cause families to fall into poverty or return to poverty due to their disabilities (9, 10). Equality in health service utilization is the basis of achieving health equality (11). A recent report demonstrated that health inequalities for persons with disabilities are influenced by multiple intersections, especially social determinants of health and broad barriers in the health system (2). Different disability types suffer various environmental and self-dysfunctional issues, which may result in more pronounced inequalities in specific subgroups (12). Research have pointed out that China currently at a medium-high level of social disability risk, suggesting that the Government urgently needs to take measures to meet the needs of the aging population and the older adult population with disabilities and semi-disabilities (13). As a result, understanding the use of health services and its inequalities for people with disabilities to support their access to health services and avoid significant complications and secondary diseases becomes an urgent issue in China.

The international policy and guidance framework for disability health inequalities has evolved over time (2). Many programs proposed in recent years, such as the WHO Global Disability Action Plan 2014–2021 (2014), the Sustainable Development Goal (SDG) target 3.8 (2015) explicitly refers to “access to health services for all persons with disabilities,” and the political declaration of “Universal health coverage” (2019) includes a specific reference for persons with disabilities. At the 74th World Health Assembly in 2021, Resolution 74.8 sets out “The highest attainable standard of health for persons with disabilities.” Furthermore, many countries have established policy promises to improve the health of people with disabilities. The Disability Strategy 2021–2031 has been formed in Australia, which includes the health and well-being of people with disabilities as one of seven outcome areas (14). To facilitate people with disabilities access to comprehensive health care, the Brazilian Ministry of Health adopted the National Health Policy for Persons with Disabilities (PNSPD) in 2002 and has recently made clear commitments to increase the availability of assistive technologies and rehabilitative services (15, 16). Due to the enormous number of disabled persons in China, the Law on the Protection of Persons with Disabilities was first implemented in 1991, which signaled the start of China’s legalization of disability protection. A unique welfare subsidy system for persons with disabilities, including living allowances for persons with difficulties and care subsidies for severely disabled persons, was established in 2015 (17). Guidance on the Expansion of the Pilot Long-term Care Insurance System (2020) proposed to focus on fullfill the fundamental care protection needs of older adults, the older adult who are disabled, and those with severe impairments (18). Medical rehabilitation projects are covered by medical insurance and expanded to 29 services. Although many nations have achieved significant progress, it is still far from ensuring people with disabilities get access to the highest attainable standard of health.

Previous studies have been carried out worldwide on the inequalities in health services utilization for persons with disabilities. According to Jeon B’s (4) analysis of the characteristics of persons with disabilities and the relationship between disability severity and health care utilization, people with disabilities have less access to preventative and outpatient health services. Julie Maltais (19) compared the healthcare utilization of intellectual disabilities to that of the general population, discovering inequalities for intellectual disabilities, particularly those more likely to develop secondary health issues. In Afghanistan, Trani Jean-Francois’s (20) analysis of health care utilization and inequalities for persons with disabilities shows inequalities in health care utilization in favor of low-income people. Several studies on health inequalities for people with disabilities have been conducted in China, some of which are based on functional disability measurements such as activities of daily living (ADL) and instrumental activities of daily living (IADL) to analyze the socioeconomic characteristics of disability in older adults in China (21, 22), and to determine the inequalities in functional disability of older populations (23). Scholars such as Xiao Jian (8) discovered disparities in health service utilization across disability trajectory categories, including progressive, late-onset, and normal categories. Guo Chao (24) investigated socioeconomic disparities in mental health service utilization among older adults with intellectual disabilities in China, finding that urban residence, higher education, marital status, health insurance coverage, and higher household income were associated with higher mental health service utilization. Their research teams also analyzed the utilization rate of auxiliary aids and healthcare services between 1987 and 2006 (25). Xintong Zhao (26) analyzed the unmet healthcare needs of people with disabilities across different residence and disability types in China and found that the rural–urban factor were significantly associated with unmet needs with rural types experiencing a significant increase in unmet healthcare needs of 13–40%. However, the older adult, people with chronic illnesses, and other demographics are primarily the focus of studies on health service utilization inequalities in China. Such as Jing Guo (27) explored socioeconomic inequalities among chronic disease populations, revealing significant differences in inequalities related to living areas, education level, economic status, and social participation. Xiaojing Fan (28) studied the inpatient health services utilization among urban and rural residents and found that the inpatient health services utilization was more concentrated among low economic groups. As a result, we reveal that inequalities exist in middle-aged and older adult persons with disabilities and may eventually worsen due to the aging population, making health service utilization inequalities for the disabled a critical public health issue.

Present research on the disabled population is insufficient since there lacks a comprehensive analysis of the utilization of health services and the inequalities in services utilization of the disabled population in Chinese. Firstly, the majority of these studies were cross-sectional in design, assessing health services inequalities at a single point in time, which fails to capture the trajectory over time. Secondly, these studies were limited to a particular type of disability or within the overall type of disability and were not subdivided into more types of disability. Thirdly, these studies have primarily examined a certain type of health services utilization. It is therefore possible to provide comprehensive evidence based on the Chinese population by analyzing each type of disability and each type of health service utilization and exploring changes over time. This study aims to analyze the utilization and the inequality of health services among middle-aged and older adult persons with disabilities and sub-types in Chinese population, and explore changes over time. Targeted references and strategies for promoting health equality and protecting the fundamental medical rights and interests of persons with disabilities were provided.

## Materials and methods

### Data sources and study design

China Health and Retirement Longitudinal Survey (CHARLS) was used in the current study. It is a longitudinal survey designed to represent the population aged 45 years and older in mainland China. The baseline survey was conducted in 2011–12, followed by wave 2 in 2013, wave 3 in 2015, and wave 4 in 2018. To ensure a representative sample, the CHARLS baseline survey covered 150 counties/districts and 450 villages/urban communities across China, involving 17,708 individuals in 10,257 households, covering the Chinese middle-aged and older adult population (29).

This study focused on middle-aged and older persons with disabilities, and the inclusion criteria were: (1) aged 45 and over. (2) had one or more disabilities. The situation of disability was extracted from the question “Do you have one of the following disabilities” in the CHARLS, which included five options: physical disabilities, intellectual disabilities, vision problems, hearing problems, and speech impediments. Participants with two or more disability problems were defined as “Multiple disabilities.”

The current utilization of health services were analyzed firstly, then the inequalities were measured and the main influences affecting service utilization and its inequalities were explored. A longitudinal comparative analysis was conducted using the four waves in CHARLS: 2011, 2013, 2015, and 2018. The data were first selected by age and type of disability inclusion criteria, obtaining 3,088, 2,388, 2,634, and 2,425 individuals, respectively. Secondly, the variable of annual *per capita* household expenditure, is an important socioeconomic status variable for this study. The distribution of the data was observed by plotting a histogram, which revealed a small number of extreme values at both ends, which may adversely affect our analyses. By referring to the literature’s treatment of the annual *per capita* household expenditure variable and combining it with the distribution of the data in this study, we chose 2 per cent as the threshold to exclude the outliers of the annual *per capita* household expenditure variable, which could effectively removes these extreme values while retaining most of the observations within the normal range. Ultimately, 2,910, 2,123, 2,442, and 2,320 persons with disabilities were chosen for the study.

### Measurements

#### Outcome variables

Three binary outcome variables of health services utilization by the disabled were adopted, outpatient service utilization, inpatient service utilization, and self-treatment service utilization. The three variables were extracted from the following questions in the CHARLS database: “In the last month have you visited a public hospital, private hospital, public health center, clinic, or health worker’s or doctor’s practice, or been visited by a health worker or doctor for outpatient care?,” “Have you received inpatient care in the past year?” and “Did you use any of the following self-treatment methods during the past month? (circle all that apply).” The answers to the questions were either “yes” or “no” and assign a value of “1” to the “yes” option and “0” to the “No” option.

#### Independent variables

In order to measure the horizontal inequity in healthcare utilization, it is essential to standardize health care needs of individuals. Actual services use is a factual depiction of the extent of equality (or inequality) in the distribution of health services. Need-expected services use represents predicted services use based on the needs-based variables. Need-standardized services use means the actual distribution of services use that is determined by non-need factors in the absence of differences in the distribution of health needs (30, 31). Therefore, study defined the health services utilization is associated not only with responses to need variables, but also with non-need variables (32). Needs-based variables are those related to the characteristics and health status that impact their medical service needs. The non-needs variables refer to the socioeconomic related factors that affect the demand for health services utilization, beyond the own health problems variable (31–33). Ideally, the needs-based variables would be the determinants of health service utilization. In this study, the Needs-based variables were defined as gender, age, self-rated health, chronic disease, activities of daily living (ADL), and instrumental activity of daily living (IADL). The non-needs variables were defined as household expenditure level, smoking, alcohol drinking, marital status, educational level, social activity, child financial support, region, and basic medical insurance.

In this study, the socioeconomic status was measured by the annual *per capita* household expenditure level, which represents household expenditure in the year preceding the survey and is the average of the permanent household population, excluding productive expenditure (31, 34). This measurement avoids the possible impact of household income outliers in the CHARLS database (35). At the same time, in order to minimize the effect of the variance in household expenditure caused by the state of the national economy in each year, the annual *per capita* household expenditures variable was divided into five levels of “Low level,” “Low-middle level,” “Average level,” “High-middle level,” and “High level” by calculating the 20 per cent, 40 per cent, 60 per cent, and 80 per cent quartiles for each of the four waves. The specific definitions of the variables are shown in Table 1.

**Table 1:** Basic information on middle-aged and older persons with disabilities, 2011–2018 *n* (%).

Variable	Category	2011	2013	2015	2018	*χ* ^2^
Participants		2910 (29.7)	2123 (21.7)	2442 (24.9)	2320 (23.7)	
Disability types	Physical disability	435 (14.9)	312 (14.7)	383 (15.7)	365 (15.7)	129.517^***^
	Intellectual disability	247 (8.5)	300 (14.1)	279 (11.4)	383 (16.5)	
	Vision problem	583 (20.0)	425 (20.0)	490 (20.1)	438 (18.9)	
	Hearing problem	955 (32.8)	653 (30.8)	801 (32.8)	662 (28.5)	
	Speech impediment	21 (0.7)	23 (1.1)	40 (1.6)	50 (2.2)	
	Multiple disabilities	669 (23.0)	410 (19.3)	449 (18.4)	422 (18.2)	
Needs-based variables						
Gender	Male	1518 (52.2)	1114 (52.5)	1212 (49.6)	1121 (48.3)	11.793^**^
	Female	1388 (47.8)	1009 (47.5)	1230 (50.4)	1199 (51.7)	
Age	45–59	1137 (39.1)	771 (36.3)	919 (37.6)	709 (30.6)	89.041^***^
	60–74	1199 (41.2)	1035 (48.8)	1170 (47.9)	1173 (50.6)	
	75 and above	574 (19.7)	317 (14.9)	353 (14.5)	438 (18.9)	
Self-rated health	Good	187 (12.6)	131 (11.9)	139 (12.0)	222 (11.0)	6.025
	General	576 (38.7)	445 (40.6)	488 (42.3)	846 (41.8)	
	Poor	725 (48.7)	521 (47.5)	527 (45.7)	955 (47.2)	
Chronic diseases	No	612 (21.0)	470 (23.0)	541 (25.5)	890 (38.4)	224.418^***^
	Yes	2298 (79.0)	1573 (77.0)	1578 (74.5)	1430 (61.6)	
ADL	No	2428 (83.5)	1775 (83.8)	2023 (82.9)	1908 (82.3)	2.218
	Yes	479 (16.5)	344 (16.2)	417 (17.1)	411 (17.7)	
IADL	No	1937 (66.6)	1321 (62.3)	1546 (63.4)	1413 (60.9)	20.159^***^
	Yes	970 (33.4)	798 (37.7)	894 (36.6)	906 (39.1)	
Non-needs variables						
Household expenditure level	Low level	585 (20.1)	409 (19.3)	470 (19.2)	464 (20.0)	1.185
	Low-middle level	583 (20.0)	430 (20.3)	492 (20.1)	464 (20.0)	
	Average level	578 (19.9)	426 (20.1)	498 (20.4)	464 (20.0)	
	High-middle level	583 (20.0)	434 (20.4)	493 (20.2)	464 (20.0)	
	High level	581 (200)	424 (20.0)	489 (20.0)	464 (20.0)	
Smoking	No	1891 (65.0)	1458 (68.7)	1773 (72.6)	1750 (75.4)	76.277^***^
	Yes	1016 (35.0)	665 (31.3)	669 (27.4)	570 (24.6)	
Alcohol drinking	No	1980 (68.2)	1401 (66.2)	1633 (67.0)	1633 (70.4)	10.159^*^
	Yes	925 (31.8)	714 (33.8)	803 (33.0)	687 (29.6)	
Marital status	Other	636 (21.9)	345 (16.3)	526 (21.5)	431 (18.6)	31.029^***^
	In marriage	2274 (78.1)	1773 (83.7)	1916 (78.5)	1889 (81.4)	
Education level	Illiterate	1127 (38.8)	548 (32.1)	630 (31.2)	683 (29.4)	80.633^***^
	Below primary school	637 (21.9)	346 (20.3)	456 (22.6)	559 (24.1)	
	Primary school	572 (19.7)	400 (23.5)	435 (21.6)	476 (20.5)	
	Junior high school and above	569 (19.6)	411 (24.1)	497 (24.6)	602 (25.9)	
Social activity	None	1494 (57.7)	915 (48.0)	1149 (50.2)	1279 (55.1)	53.084^***^
	Yes	1096 (42.3)	990 (52.0)	1140 (49.8)	1041 (44.9)	
Child financial support	No	949 (55.3)	173 (17.2)	250 (17.7)	171 (13.7)	882.255^***^
	Yes	766 (44.7)	833 (82.8)	1163 (82.3)	1080 (86.3)	
Region	Western Region	1096 (37.7)	913 (43.0)	917 (37.6)	896 (38.6)	22.723^***^
	Central Region	992 (34.1)	667 (31.4)	883 (36.2)	794 (34.2)	
	Eastern Region	822 (28.2)	543 (25.6)	642 (26.3)	630 (27.2)	
Basic medical insurance	None	230 (7.9)	109 (5.2)	243 (10.2)	97 (4.2)	80.253^***^
	Yes	2669 (92.1)	1995 (94.8)	2140 (89.8)	2221 (95.8)	

^*^p value < 0.05, ^**^p value < 0.01, ^***^p value < 0.001.

#### Data analysis

Descriptive analysis was shown by frequency and percentage. Chi-squared test was used to analyze the differences in sample characteristics, health services utilization across waves and disability types. The wave factors were included in the pooled cross-section regression to evaluate the associated factors with health services utilization. The *p*-values, odd ratio (OR) and 95% confidence interval were reported. The health services utilization inequalities were measured by the Concentration index (CI), Horizontal inequity index (HI) and decomposition of concentration index (36). The STATA 14.0 was used for data analysis.

### Concentration index

The Concentration index (CI) was used to measure the inequities in health services utilization, which was introduced by Wagstaff (36) to measure income-related health service use and health inequities and is widely accepted. CI ranges from −1 to 1, with the positive value indicating that income-related inequality concentrates on the rich, whereas the negative value indicates pro-poor inequality. A zero value represents that the distribution of healthcare utilization is equal. The equation of CI is as follows (37):


$$C=\frac{2}{\mu }cov\left(h,r\right)$$

Where C was defined in terms of the covariance between the outcome variable (*h*) for whether health service utilization occurs and the fractional ranks of annual *per capita* household consumption expenditure (*r*). *μ* is the mean value of health service utilization (*h*).

### Horizontal inequity index

The Horizontal inequity index (HI) measured the utilization of necessary standardized health services. It reveals inequalities in health service utilization by controlling for the influence of need-based variables on health service use. It is interpreted as the impact on health service utilization due to different socioeconomic status when the health condition is given the same demand for health services (38). Similar to CI, the positive value suggests the health service is more concentrated on the wealthier groups and vice versa. The equation of HI is as follows:


$$HI=CI-\sum j\left(\beta_{j}^{m}\bar{x_{j}}/\mu \right)c_{j}$$

Where $x_{j}$ is the needs-based variables, $\beta_{j}$ is the marginal effects (*dy/dx*) of $x_{j}$ , $\bar{x_{j}}$ is the means of $x_{j}$ , $c_{j}$ is the concentration index of $x_{j}$ .

### Decomposition of inequality

The method of decomposition of the Concentration index, proposed by Wagstaff et al., was used to analyze the contribution of independent variables to the inequalities (39). A Probit regressions model was employed to calculate the effects. A positive concentration index indicates that the factor exacerbates inequality in the use of health services, and a negative concentration index indicates that it reduces inequality. The percentage contribution rate represents the factor’s contribution extent to inequity. The equation is as follows:


$$C=\underset{j}{\sum }\left(\beta_{j}^{m}\bar{x_{j}}/\mu \right)c_{j}+\underset{k}{\sum }\left(\gamma_{k}^{m}\bar{z_{k}}/\mu \right)c_{k}+GC\varepsilon /\mu$$

$z_{k}$ is the non-need variable, $\gamma_{k}$ is the marginal effects (dy/dx) of $z_{k}$ , $\bar{z_{k}}$ is the means of $z_{k}$ , $c_{j}$ , and $c_{k}$ are the concentration indexes of $x_{j}$ and $z_{k}$ , GCε is the concentration index of the error term *ε*.

#### Ethical issues/statement

Ethical approval was obtained from the Institutional Review Board of Peking University (protocol code IRB00001052-11015) for the collection of human subjects data. All participants provided informed consent before data collection.

## Results

### Basic information for persons with disabilities

Table 1 shows the basic information about middle-aged and older persons with disabilities. A total of 9,795 individuals were included in the study. The majority of people had hearing problems, followed by vision problems and multiple disabled. The age increased during the 4 years, predominantly aged 60–74, accounting for 41.2, 48.8, 47.9, and 50.6%, respectively. Health status is predominantly poor, with more than 60% of the population suffering from chronic diseases. Age, IADL, education, child financial support, and basic medical insurance were statistically significant (*p* < 0.05) in the comparison of 4 year.

### Health services utilization for persons with disabilities

The utilization of health services for persons with disabilities was demonstrated in Table 2. There were statistically significant differences in outpatient, inpatient, and self-treatment service utilization between years (*p* < 0.05). Over the 4 years, outpatient service utilization was 23.4, 28.1, 25.4, and 20.7%, with non-utilization decreasing year on year from 87.9% (2011) to 79.0% (2015). Persons with multiple disabilities used outpatient services the most (27.1%), whereas speech impediments had the greatest non-utilization rate (87.5%). The hospitalization rate grew from 12.1% in 2011 to 27% in 2018, although the no-hospitalization rate increased from 7.9% in 2011 to 27.0% in 2018. Persons with speech impediments had the highest rates of hospitalization and no-hospitalization, followed by people with multiple disabilities. There has been a decrease in the rate of outpatient services that should have been seen but not seen, from 88.7 to 79%. The proportion of should have been hospitalized but were not has shown an increase and then decrease. Self-treatment utilization increased from 55.6% (2011) to 68.3% (2018), with intellectual disability (64.6%) having the highest prevalence, followed by multiple disabilities (63.8%).

**Table 2:** Health service utilization for persons with disabilities *n* (%).

Variable	Waves				Type of disability					
	2011	2013	2015	2018	Physical disability	Intellectual disability	Vision problem	Hearing problem	Speech impediment	Multiple disabilities
Outpatient services										
Outpatient service utilization				36.087^***^						27.393^***^
Yes	677 (23.4)	594 (28.1)	619 (25.4)	480 (20.7)	328 (22.0)	289 (24.1)	499 (25.8)	713 (23.3)	16 (12.0)	525 (27.1)
No	2215 (76.6)	1517 (71.9)	1816 (74.6)	1838 (79.3)	1161 (78.0)	912 (75.9)	1433 (74.2)	2349 (76.7)	117 (88.0)	1414 (72.9)
Should have been seen but not seen				12.274^**^						1.943
Yes	333 (87.9)	282 (85.2)	339 (79.0)	—	145 (85.8)	95 (85.6)	215 (83.3)	241 (81.7)	14 (87.5)	244 (84.1)
No	46 (12.1)	49 (14.8)	90 (21.0)	—	24 (14.2)	16 (14.4)	43 (16.7)	54 (18.3)	2 (12.5)	46 (15.9)
Inpatient services										
Inpatient service utilization				194.602^***^						104.217^***^
Yes	352 (12.1)	447 (21.1)	548 (22.5)	627 (27.0)	297 (19.9)	301 (24.9)	360 (18.6)	478 (15.6)	40 (30.1)	498 (25.6)
No	2555 (87.9)	1673 (78.9)	1889 (77.5)	1691 (73.0)	1197 (80.1)	907 (75.1)	1575 (81.4)	2591 (84.4)	93 (69.9)	1445 (74.4)
Should have been hospitalized but were not				17.680^***^						62.364^***^
Yes	203 (7.9)	194 (11.5)	262 (10.7)		208 (15.2)	191 (17.2)	236 (13.1)	306 (10.8)	26 (21.1)	319 (17.9)
No	2356 (92.1)	1500 (88.5)	2177 (89.3)		1162 (84.8)	919 (82.8)	1559 (86.9)	2525 (89.2)	97 (78.9)	1462 (82.1)
Self-treatment										
Self-treatment utilization				95.511^***^						9.861
Yes	1615 (55.6)	1350 (64.1)	1529 (63.1)	1584 (68.3)	902 (60.6)	778 (64.6)	1217 (63.0)	1867 (61.0)	78 (58.6)	1236 (63.8)
No	1291 (44.4)	755 (35.9)	896 (36.9)	734 (31.7)	587 (39.4)	426 (35.4)	714 (37.0)	1193 (39.0)	55 (41.4)	701 (36.2)

^*^p value < 0.05, ^**^p value < 0.01, ^***^p value < 0.001.

### Pooled cross-section regression of the health services utilization

The results of the pooled cross-section regressions were shown in Table 3, where there was an increase in the utilization of inpatient and self-treatment services from 2013 to 2018 compared to 2011 (*p* < 0.05). Older people utilized more inpatient and self-treatment than middle-aged people with disabilities. The inpatient service utilization was higher for male, and the self-treatment service utilization was higher for female. In the health-related factors, those with poorer self-rated health and with chronic diseases had higher utilization of the three health services, while those with ADL (OR = 1.365) and IADL (OR = 1.733) had higher utilization of inpatient services. Household expenditure level contributes to outpatient and inpatient service utilization. High levels of education facilitated inpatient and self-treatment services utilization (*p* < 0.05). Socially active people had 1.362 times higher utilization of outpatient services and 1.18 times higher self-treatment than non-socially active people. Having medical insurance promoted the utilization of inpatient services (OR = 2.115). After adding the disability types variable to the three types of service use, the results showed that factors have a stable effect on the utilization. With regard to the category of disability, only the results in outpatient services utilization indicated that individuals with multiple disabilities exhibited a higher utilization rate than those with physical disability (OR = 1.436). The other results had no significant effect (*p* > 0.05).

**Table 3:** Pooled cross-section regression of the health services utilization for persons with disabilities.

Variables	Outpatient service utilization		Inpatient service utilization		Self-treatment utilization	
	Odds ratio	Odds ratio	Odds ratio	Odds ratio	Odds ratio	Odds ratio
Disability types: Intellectual disability (Reference: Physical disability)		1.161		1.080		1.223
		(0.209)		(0.197)		(0.195)
Vision problem		1.212		0.997		1.020
		(0.190)		(0.167)		(0.138)
Hearing problem		1.230		0.741*		1.210
		(0.181)		(0.119)		(0.150)
Speech impediment		0.844		0.808		1.410
		(0.407)		(0.392)		(0.641)
Multiple disabilities		1.436**		1.080		1.045
		(0.223)		(0.175)		(0.144)
Wave: 2013 (Reference: 2011)	1.428**	1.425**	1.879***	1.850***	1.503***	1.481***
	(0.214)	(0.215)	(0.344)	(0.340)	(0.205)	(0.203)
2015	1.044	1.057	1.771***	1.763***	1.461***	1.443***
	(0.149)	(0.151)	(0.295)	(0.295)	(0.183)	(0.181)
2018	0.845	0.857	2.400***	2.347***	1.927***	1.908***
	(0.109)	(0.112)	(0.359)	(0.354)	(0.216)	(0.216)
Gender: Female (Reference: Male)	1.101	1.095	0.746**	0.749**	1.406***	1.409***
	(0.128)	(0.128)	(0.0931)	(0.0937)	(0.145)	(0.146)
Age: 60–74 (Reference: 45–59)	1.123	1.111	1.380***	1.403***	1.352***	1.337***
	(0.125)	(0.124)	(0.168)	(0.171)	(0.131)	(0.130)
75 and above	0.944	0.923	1.521**	1.562***	1.253*	1.239
	(0.149)	(0.147)	(0.251)	(0.259)	(0.169)	(0.168)
Self-rated health: General (Reference: Good)	1.789***	1.778***	1.338	1.352	1.537***	1.530***
	(0.361)	(0.359)	(0.287)	(0.290)	(0.208)	(0.207)
Pool	3.258***	3.237***	2.242***	2.208***	2.081***	2.087***
	(0.655)	(0.654)	(0.474)	(0.466)	(0.289)	(0.290)
Chronic diseases: Yes (Reference: No)	1.366***	1.347***	1.464***	1.432***	1.858***	1.862***
	(0.152)	(0.151)	(0.178)	(0.176)	(0.171)	(0.173)
ADL: Yes (Reference: No)	1.017	1.035	1.365**	1.322*	1.237	1.271*
	(0.143)	(0.147)	(0.192)	(0.190)	(0.176)	(0.183)
IADL: Yes (Reference: No)	1.005	0.994	1.733***	1.692***	1.142	1.148
	(0.112)	(0.111)	(0.202)	(0.197)	(0.120)	(0.121)
Household expenditure level: Low-middle level (Reference: Low level)	1.380**	1.373**	1.435*	1.416*	1.022	1.027
	(0.210)	(0.210)	(0.266)	(0.264)	(0.129)	(0.130)
Average level	1.693***	1.688***	2.696***	2.670***	1.198	1.204
	(0.252)	(0.252)	(0.471)	(0.466)	(0.153)	(0.154)
High-middle level	1.546***	1.544***	2.441***	2.410***	1.244*	1.252*
	(0.234)	(0.235)	(0.421)	(0.418)	(0.159)	(0.161)
High level	1.751***	1.746***	3.895***	3.848***	1.227	1.227
	(0.264)	(0.264)	(0.666)	(0.661)	(0.158)	(0.158)
Smoking: Yes (Reference: No)	0.798**	0.801*	0.602***	0.604***	1.013	1.009
	(0.0918)	(0.0925)	(0.0783)	(0.0790)	(0.101)	(0.101)
Alcohol drinking: Yes (Reference: No)	0.832*	0.828*	0.740**	0.743**	0.975	0.971
	(0.0904)	(0.0905)	(0.0902)	(0.0907)	(0.0924)	(0.0923)
Marital status: Married (Reference: Other)	1.171	1.179	0.940	0.941	1.081	1.085
	(0.133)	(0.135)	(0.111)	(0.112)	(0.108)	(0.109)
Education level: Below primary school (Reference: Illiterate)	0.790*	0.786*	1.359**	1.341**	1.243*	1.242*
	(0.0996)	(0.0995)	(0.189)	(0.186)	(0.144)	(0.144)
Primary school	0.669***	0.666***	1.218	1.217	1.414***	1.411***
	(0.0913)	(0.0909)	(0.184)	(0.184)	(0.174)	(0.174)
Junior high school and above	0.853	0.860	1.267	1.254	1.853***	1.847***
	(0.122)	(0.124)	(0.203)	(0.201)	(0.239)	(0.238)
Social activities: Yes (Reference: No)	1.362***	1.362***	1.015	1.020	1.180**	1.181**
	(0.127)	(0.127)	(0.104)	(0.105)	(0.0986)	(0.0989)
Child support: Yes (Reference: No)	1.179	1.182	1.288*	1.289*	1.001	1.000
	(0.145)	(0.145)	(0.176)	(0.176)	(0.105)	(0.105)
Region: Central Region (Reference: Western Region)	0.849	0.846	0.863	0.859	0.828**	0.823**
	(0.0898)	(0.0896)	(0.0963)	(0.0960)	(0.0788)	(0.0784)
Eastern Region	0.943	0.956	0.723**	0.735**	0.887	0.879
	(0.108)	(0.110)	(0.0936)	(0.0960)	(0.0925)	(0.0921)
Basic medical insurance: Yes (Reference: No)	1.329	1.324	2.115***	2.154***	1.309	1.298
	(0.264)	(0.263)	(0.512)	(0.518)	(0.215)	(0.214)
Constant	0.0490***	0.0411***	0.0103***	0.0112***	0.165***	0.152***
	(0.0167)	(0.0147)	(0.00414)	(0.00464)	(0.0453)	(0.0437)
*R* ^2^	0.0587	0.0607	0.1270	0.1301	0.0586	0.0598

Robust standard errors in parentheses, ***p < 0.01, **p < 0.05, *p < 0.1.

### Inequalities analysis for health services utilization

**Figure 1:**
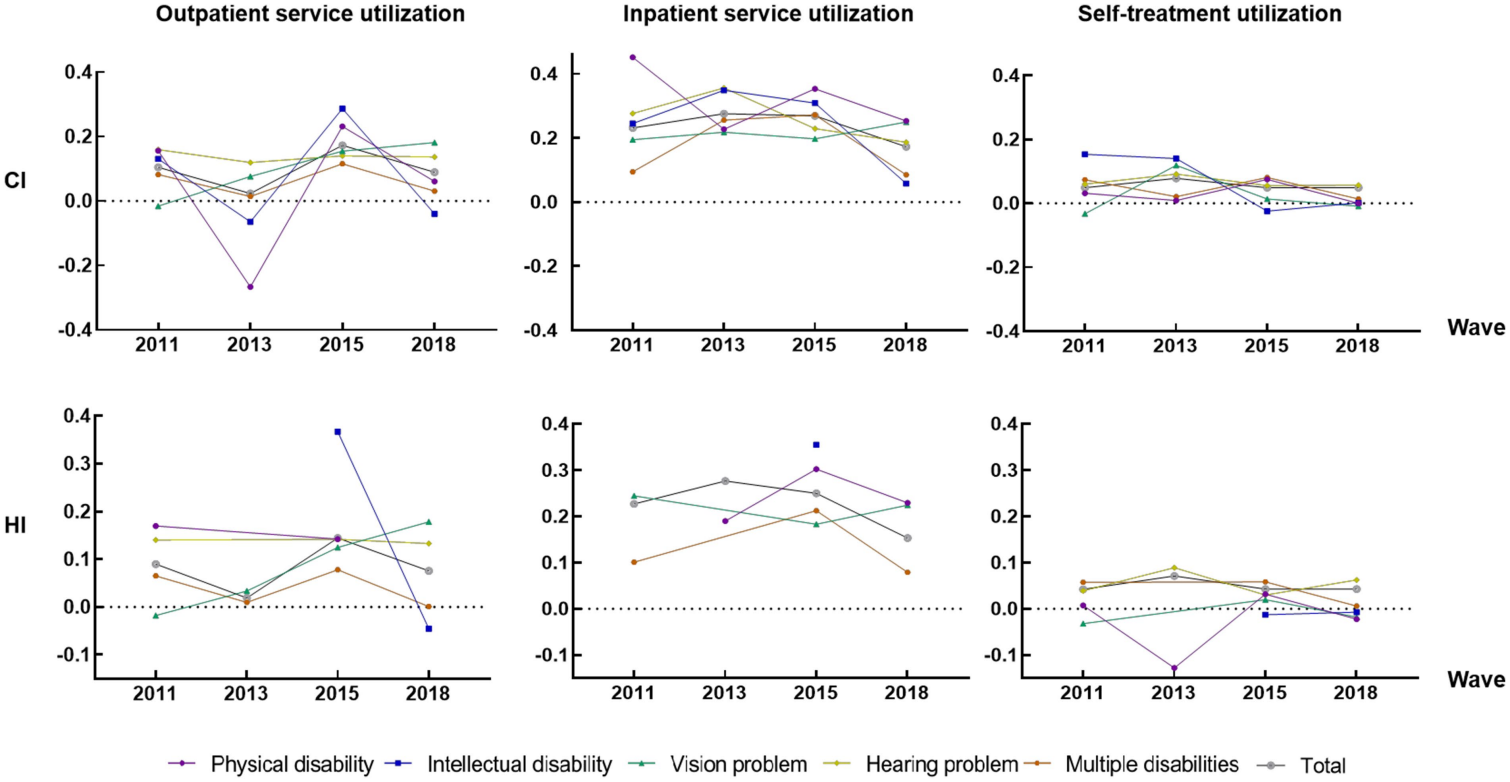
Inequality analysis for health service utilization by different waves and disabilities groups. HI results have some missing values due to insufficient data quantity.

**Figure 2:**
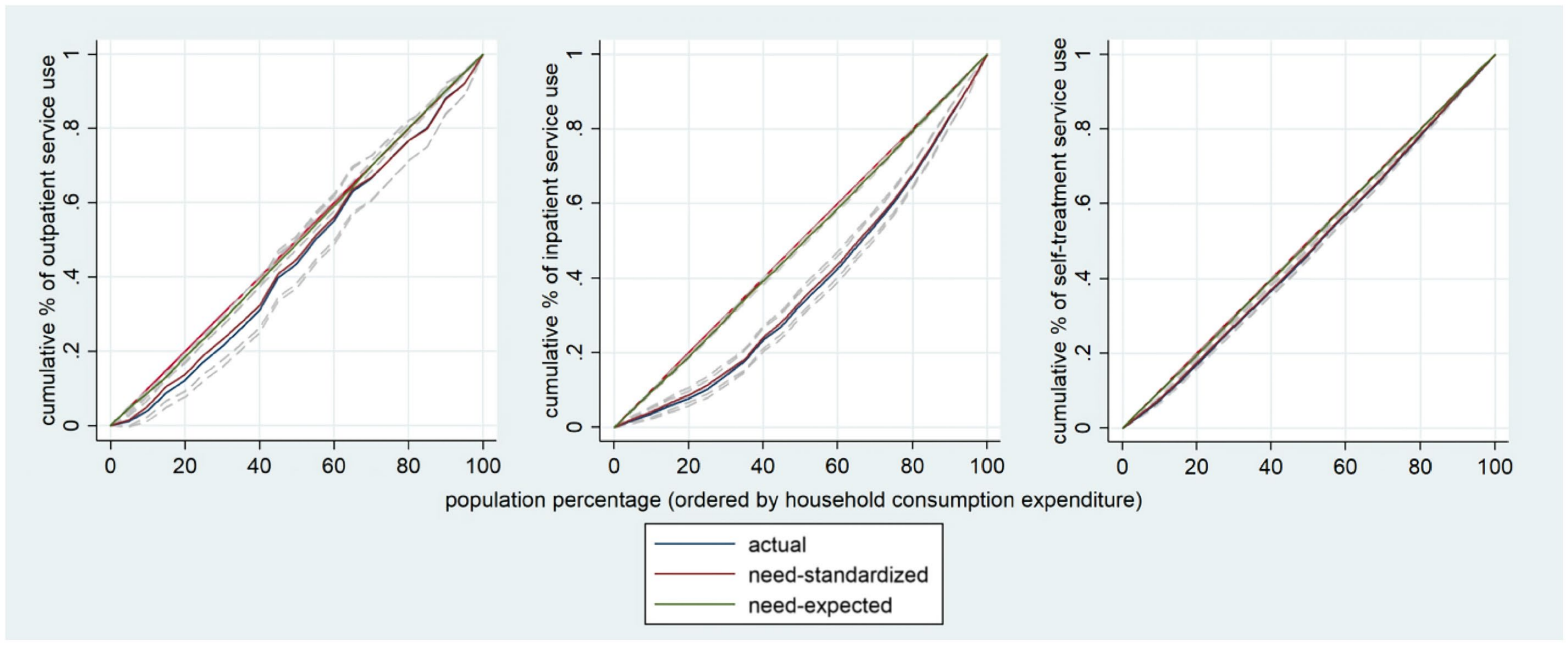
Concentration curve for health service use by disabled people.

Figure 1 illustrated the CIs and the HIs for health services utilization from 2011 to 2018, with all positive results for inpatient services (*p* < 0.01), indicating that there were pro-rich inequalities in the services use caused by socioeconomic related characteristics. Among the three types of health service utilization, inpatient service utilization had the highest degree of inequality (0.2480 for CI in all groups), followed by outpatient service utilization (0.0882 for CI in all groups) and self-treatment (0.0487 for CI in all groups) (Figure 2). Among the annual differences, as a whole, the inpatient service utilization decreased while the use of self-treatment service utilization essentially constant. The biggest disparities of utilization of inpatient services were for physical disabilities (0.4515 for CI in 2011), and the highest disparities of utilization of self-treatment services were for intellectual disability (0.1538 for CI in 2011).

### Decomposition of inequalities in health services utilization

Table 4 analyzed the decomposition of inequality in health service utilization for 2011 and 2018, revealing that expenditure is the most significant contribution to inequalities in health services utilization, followed by self-rated health status, contributing to pro-rich inequalities. The annual trend showed a gradual decrease in expenditure contribution, from 112.87 to 65.06% in outpatient utilization and from 74.82 to 47.73% in self-treatment service utilization. The inequalities were also influenced by chronic disease, educational level and health insurance factors.

**Table 4:** Decomposition of inequalities in health services utilization in 2011 and 2018.

Type	Outpatient service utilization				Inpatient service utilization				Self-treatment utilization			
	2011		2018		2011		2018		2011		2018	
	Concentration Index	Contribution rate (%)	Concentration Index	Contribution rate (%)	Concentration Index	Contribution rate (%)	Concentration Index	Contribution rate (%)	Concentration Index	Contribution rate (%)	Concentration Index	Contribution rate (%)
Gender: Female	0.0292	0.46%	0.0275	−3.22%	0.0304	0.22%	0.0275	−2.21%	0.0304	1.65%	0.0275	5.64%
Age: 60–74	−0.0259	−2.19%	−0.0263	−0.41%	−0.0273	−1.99%	−0.0263	−0.93%	−0.0273	−4.00%	−0.0263	−10.35%
75 and above	−0.0899	−1.24%	−0.0291	0.75%	−0.0886	−5.07%	−0.0291	−1.11%	−0.0886	−5.99%	−0.0291	−3.27%
Self-rated health: General	−0.0216	−2.17%	−0.0274	−7.28%	−0.0232	−1.87%	−0.0274	0.26%	−0.0232	−0.17%	−0.0274	−12.76%
Pool	0.0490	17.59%	0.0526	24.29%	0.0502	6.42%	0.0526	6.43%	0.0502	12.68%	0.0526	31.32%
Chronic diseases: Yes	0.0244	7.07%	0.0261	2.52%	0.0240	1.44%	0.0261	2.79%	0.0240	14.93%	0.0261	18.00%
ADL: Yes	0.0284	0.11%	0.1281	−0.46%	0.0296	0.60%	0.1281	2.92%	0.0296	0.67%	0.1281	2.22%
IADL: Yes	0.0610	−5.08%	0.0312	1.14%	0.0622	2.40%	0.0312	2.23%	0.0622	1.30%	0.0312	4.68%
Household expenditure	0.1225	112.87%	0.1013	65.06%	0.1228	93.96%	0.1013	93.91%	0.1228	74.82%	0.1013	47.73%
Smoking: Yes	−0.0360	2.69%	−0.0583	6.25%	−0.0348	2.52%	−0.0583	1.81%	−0.0348	0.68%	−0.0583	3.47%
Alcohol consumption: Yes	−0.0807	−1.20%	0.0070	−0.93%	−0.0794	−1.88%	0.0070	−0.33%	−0.0794	3.99%	0.0070	0.18%
Marital status: Married	0.0519	4.84%	0.0012	−0.01%	0.0530	0.76%	0.0012	−0.06%	0.0530	14.33%	0.0012	0.27%
Educational level: Below primary school	−0.0698	2.00%	−0.0132	0.60%	−0.0685	−0.17%	−0.0132	−0.49%	−0.0685	−3.80%	−0.0132	0.55%
Primary school	−0.0221	2.12%	−0.0482	2.11%	−0.0209	0.02%	−0.0482	−1.35%	−0.0209	−1.18%	−0.0482	−5.62%
Junior high school and above	0.2035	−8.04%	0.1311	−4.10%	0.1970	6.80%	0.1311	2.67%	0.1970	11.65%	0.1311	32.00%
Social activities: Yes	−0.0039	−0.37%	0.0526	5.02%	−0.0026	0.02%	0.0526	−0.54%	−0.0026	−0.41%	0.0526	−3.67%
Child support: Yes	−0.0535	−4.45%	−0.0144	0.88%	−0.0547	−1.98%	−0.0144	−1.86%	−0.0547	0.41%	−0.0144	−4.13%
Region: Central Region	0.0146	0.47%	−0.0317	0.88%	0.0158	−0.80%	−0.0317	0.02%	0.0158	0.52%	−0.0317	8.61%
Eastern Region	−0.0029	−0.10%	0.0800	0.22%	−0.0016	0.11%	0.0800	−4.20%	−0.0016	−0.12%	0.0800	−14.08%
Medical insurance: Yes	0.0215	−5.84%	0.0034	0.10%	0.0213	5.27%	0.0034	0.52%	0.0213	−0.22%	0.0034	2.47%
CI	0.1103		0.0683		0.2419		0.1558		0.0470		0.0125	
HI	0.0898		0.0743		0.2262		0.1550		0.0369		0.0119	

## Discussion

Reducing health inequalities in health services utilization for persons with disabilities is necessary for promoting health equalities and social equality. Using data from four waves of CHARLS 2011, 2013, 2015 and 2018, this longitudinal comparative study focused on disability types to carry out comparisons of health service utilization and the socioeconomic related disparities in health service utilization among persons with disabilities and the sub-populations. This study provides a comprehensive analysis of health service utilization among the disabled population in China, including the utilization of health services for different periods and types of disabilities and measures the inequalities of service utilization. The national database is also used to reflect the utilization of health services in China, making the study more representative.

The utilization of health services by people with disabilities revealed poor initiative. The decrease in outpatient services utilization and the increase in self-treatment services utilization for the four-year comparison can be assumed that middle-aged and older adults with disabilities may passively avoid medical treatment and wait for self-healing facing minor illnesses due to financial and medical access difficulties. The proportion of people who “should have been hospitalized but were not” grew and is higher than the 10.2% reported in the 6th China Health Services Statistical Survey (CHSSS) in 2018 (40). It may indicate that there are still many disabled people in China who require hospitalization but the accessibility is inadequate. The World Report on Disability stated that the affordability of health services and transportation are two main barriers for people with disabilities to access health services (41). Related research found that telemedicine has the potential to be used to improve the health of the impaired population while being efficient and affordable (42). The advantages of telemedicine should therefore be fully exploited by enhancing the accessibility that considers the disabled special usability needs (43). At the same time, family carers (i.e., relatives or friends) play a key role in supporting people with disabilities to ensure that their basic needs are met while their rights are respected and protected, so the supportive role of the family can be fully realized (44).

Our finding revealed a pro-rich inequalities in health services utilization for persons with disabilities, meaning that persons with higher socioeconomic related status is more accessible to health services, consistent with the findings of Bin Guo et al. (45) on equality in urban and rural health service utilization and Sun et al. (46) on inequality in inpatient service utilization. The CIs and HIs in outpatient and inpatient services showed declines over the four waves comparisons and the pooled cross-section regression concluded that inpatient service utilization had increased in all years compared to 2011. This result is probably due to several effective measures implemented in recent years to ensure the use of health services for persons with disabilities, such as increasing the participation rate of basic medical insurance and fully covering the nursing care subsidy for key groups of persons with disabilities (47). Inpatient health service utilization inequality was the greatest of the three types of utilization, consistent with the findings of Sun et al. (46) that inpatient service utilization inequality was higher than outpatient service utilization. The severity of inpatient service utilization inequality has been highlighted in other studies (48, 49). Persons with disabilities face more health and socioeconomic risks than non-disabled people. Since the inpatient costs are much higher than outpatient and self-treatment, low-income residents are more likely to be forced into poverty (49). To ensure that patients with serious impaired and the dirt-poor have access to essential medical services, medical assistance for persons with disabilities should be upgraded and made available to key populations.

Disparities in different disability groups should also be considered. Severe inequalities were found in outpatient and self-treatment among people with intellectual disabilities. On the one hand, patients with intellectual impairments may only interact with clinicians using simple phrases, leading to misdiagnosis and delays in receiving the best care. On the other hand, there are issues with prejudice and unfavorable attitudes of doctors toward the disabled (1). Physical disabilities experience access disparities in inpatient service, which may be related to the significant demand for rehabilitation during hospitalization because of the patients’ restricted physical function and activity (50). The lengthy rehabilitation process and high rehabilitation expense considerably raise the financial burden on families of people with disabilities. Therefore, it is crucial to refine the provision of basic medical services for the individual demands of varied disabilities. Furthermore, the provision of disability-friendly health facilities and equipment would contribute to inclusive and accessible health care for all (51). However, as mentioned in the Disability Rights and Protection Act 2013 (52), using audio, braille and sign language interpreters when necessary is not implemented in many hospitals. Therefore, hospitals should improve basic infrastructure (e.g., toilets and ramps) and information and services (e.g., provision of sign language interpreters) (53) to enhance the access and well-being of people with disabilities in the health services utilization.

The economic factors were shown to be the dominant factor contributing to disparities for persons with disabilities. This may be because of the direct relationship between economic situations and health service affordability, which is a crucial element of equal access to health services (54). Consistent with the results in the utilization of preventive health services and outpatient inpatient services (46, 55). Health-related variables such as self-rated health and chronic disease also contribute to service utilization inequality, showing that service demand is the fundamental factor for service utilization. Disabled people are likely to have overlapping functional limitations or complex health conditions that increase their healthcare needs and costs (56). Attention should be paid to the chronic diseases of the middle-aged and older adult disabled population to prevent the significant financial burden of multiple co-morbidities on disabled people. Furthermore, the educational level and basic health insurance were the following factors to promote service utilization and contributed to pro-rich inequalities in socioeconomic variables. The education variable may be because persons with higher educational levels are more health-conscious and have advantages in health information access, which increases the use of health services (57, 58). In order to increase the health literacy of the disabled population and inhabitants, health education for those with low educational attainment should be a priority. Efforts to assimilate e-health literacy are important to improve the capacity for adaptation to a digital society. On this basis, the digital tools in the healthcare system need to be ensured to be suitable for persons with disabilities (59). The results of the basic health insurance factor are consistent with prior studies (60), which demonstrated that the increase in medical insurance coverage for hospitalization service expenditure promotes the utilization of health services. Consequently, a multi-level medical security system for disabled people should be established through the triple system of basic medical insurance, critical diseases insurance and medical assistance so as to improve the medical security capacity for persons with disabilities (61).

Our study also has several limitations. Firstly, reporting bias is unavoidable in the open databases used in this study based on questionnaires. Secondly, the method of concentrated index decomposition is descriptive analysis. The causal analysis and results should be interpreted with caution. Thirdly, the selection of variables was limited by the open databases. Therefore some variables that are theoretically more relevant to the utilization of services for persons with disabilities were not included. Despite the above limitations, facing the population aging with disabilities and the high social disability risk in China, attention should be paid to research on the health economics evaluation related to the utilization of services for the disabled and its subgroups.

## Conclusion

Our results indicated that the persons with disabilities are less likely to take the initiative to use health services. The pro-rich inequalities existed in health services utilization from 2011 to 2018 waves, with the disparities in inpatient services being the largest, although the inequalities decreased over time. The decomposition analysis revealed that the economy was the dominant factor exacerbating inequalities. Policies should promote medical assistance for key disabled people, improve the capacity of the primary hospital to provide medical and rehabilitation services to people with disabilities and support accessible facilities and services for different disability groups.

## Data Availability

Publicly available datasets were analyzed in this study. Data were obtained from the China Health and Retirement Longitudinal Study and are available at https://charls.pku.edu.cn/, with the permission of Peking University of China. The data underlying this article will be shared on reasonable request to the corresponding author.
